# Host specificity of two pollinating seed‐consuming fly species is not related to soil moisture of host plant in the high Himalayas

**DOI:** 10.1002/ece3.2644

**Published:** 2016-12-30

**Authors:** Bo Song, Jürg Stöcklin, Yong‐Qian Gao, De‐Li Peng, Hang Sun

**Affiliations:** ^1^ Key Laboratory for Plant Diversity and Biogeography of East AsianKunming Institute of BotanyChinese Academy of SciencesKunmingYunnanChina; ^2^ Institute of BotanyUniversity of BaselBaselSwitzerland; ^3^ Yunnan Forestry Technological CollegeKunmingYunnanChina

**Keywords:** *Bradysia*, host specificity, offspring performance, *Rheum*, soil moisture

## Abstract

Studying the drivers of host specificity can contribute to our understanding of the origin and evolution of obligate pollination mutualisms. The preference–performance hypothesis predicts that host plant choice of female insects is related mainly to the performance of their offspring. Soil moisture is thought to be particularly important for the survival of larvae and pupae that inhabit soil. In the high Himalayas, *Rheum nobile* and *R. alexandrae* differ in their distribution in terms of soil moisture; that is, *R. nobile* typically occurs in scree with well‐drained soils, *R. alexandrae* in wetlands. The two plant species are pollinated by their respective mutualistic seed‐consuming flies, *Bradysia* sp1. and *Bradysia* sp2. We investigated whether soil moisture is important for regulating host specificity by comparing pupation and adult emergence of the two fly species using field and laboratory experiments. Laboratory experiments revealed soil moisture did have significant effects on larval and pupal performances in both fly species, but the two fly species had similar optimal soil moisture requirements for pupation and adult emergence. Moreover, a field reciprocal transfer experiment showed that there was no significant difference in adult emergence for both fly species between their native and non‐native habitats. Nevertheless, *Bradysia* sp1., associated with *R. nobile*, was more tolerant to drought stress, while *Bradysia* sp2., associated with *R. alexandrae*, was more tolerant to flooding stress. These results indicate that soil moisture is unlikely to play a determining role in regulating host specificity of the two fly species. However, their pupation and adult emergence in response to extremely wet or dry soils are habitat‐specific.

## 1 Introduction

Current estimates of insect diversity range as high as 30 million species, and a large fraction of these species feed on plants (Jaenike, 1990). Furthermore, most species of phytophagous insects are highly host specific, feeding on only a small number, or even single plant species (Fry, 1996), probably because specialization has greater overall advantages than polyphagy (Bernays & Graham, 1988). Classic examples of host specificity in insects are obligate pollination mutualisms, in which a plant species is pollinated exclusively by an obligate seed‐parasitic pollinator, such as *Ficus‐Agaonidae* (Janzen, 1979), *Yucca‐Tegeticula* (Pellmyr, Thompson, Brown, & Harrison, 1996), and *Glochidion‐Epicephala* (Kato, Takimura, & Kawakita, 2003). Studying the reasons that drive such exclusive host specialization in these plant–insect interactions could contribute to our understanding of the origin and evolution of obligate pollination mutualisms (Jaenike, 1990), and have been the subject of considerable debate and speculation for decades (Ehrlich & Raven, 1964; Rausher, 1984; Yang, Li, Peng, & Yang, 2012).

In many previous studies, the evolution of host specificity was assumed to be governed by trade‐offs in the performance of adult insects and their offspring (i.e., the ability to survive and successfully develop and reproduce) on different host plants (Fry, 1996; Joshi & Thompson, 1995; Singer, 2000). However, the preference–performance hypothesis predicts that host plant choice of female insects is related mainly to the performance of their offspring, particularly during the immature stage, for example, egg hatch, pupation, and adult emergence, because of immobility (Gripenberg, Mayhew, Parnell, & Roslin, 2010; Jaenike, 1990). It is generally assumed that offspring performance on a specific plant species depends on several ecological variables, including nutritional suitability, natural enemies of insects, and the abiotic environmental conditions (Martin & Pullin, 2004; Slansky, 1993). Of these factors, abiotic environmental conditions have been found to be most important in determining host specialization of insect species in several cases, especially among related plant species with similar chemical, phenological, and physical characteristics (Arvanitis, Wiklund, & Ehrlén, 2007, 2008; Chew & Robbins, 1984; Courtney, 1986; Dempster, 1983; König, Wiklung, & Ehrlén, 2016; Martin & Pullin, 2004; Whittaker & Feeny, 1971). For example, Martin and Pullin (2004) showed that the specialization of the butterfly *Lycaena dispar* (Lepidoptera: Lycaenidae) on *Rumex hydrolapathum* is mainly determined by conditions of the abiotic environment and not by characteristics of the host plant itself.

Soil moisture is one of the most important environmental factors determining offspring performance in insects, particularly for the developmental and natural mortality rates of soil‐dwelling insect stages (Johnson, Zhang, Crawford, Gregory, & Young, 2007), and thus regulating the population establishment and abundance of insects (Eskafi & Fernandez, 1990). On the one hand, high levels of soil moisture can induce a lack of oxygen in the soil, limiting pupation and adult eclosion, and may even cause death of larvae and pupae (Eskafi & Fernandez, 1990; Hulthen & Clarke, 2006). On the other hand, low levels of soil moisture can cause desiccation, a predominant cause of larval and pupal mortality in many insect species (Bressan‐Nascimento, 2001; Hou, Xie, & Zhang, 2006). For example, extremely wet or dry soils significantly hindered pupating and eclosion in *Contarinia nasturtii* (Diptera: Cecidomyiidae) (Chen & Shelton, 2007). Nevertheless, insect species can be adapted to different ranges of soil moisture. For example, pupae of *Anastrepha oblique* are less tolerant of dry soils than *A. ludens* (Montoya, Flores, & Toledo, 2008). Thus, according to the preference–performance hypothesis, the range of soil moisture at which a plant species occurs may influence the oviposition preference of adult flies on this plant species for maximizing the development and survival of their offspring with low mobility (Bonebrake, Boggs, McNally, Ranganathan, & Ehrlich, 2010).

**Figure 1:**
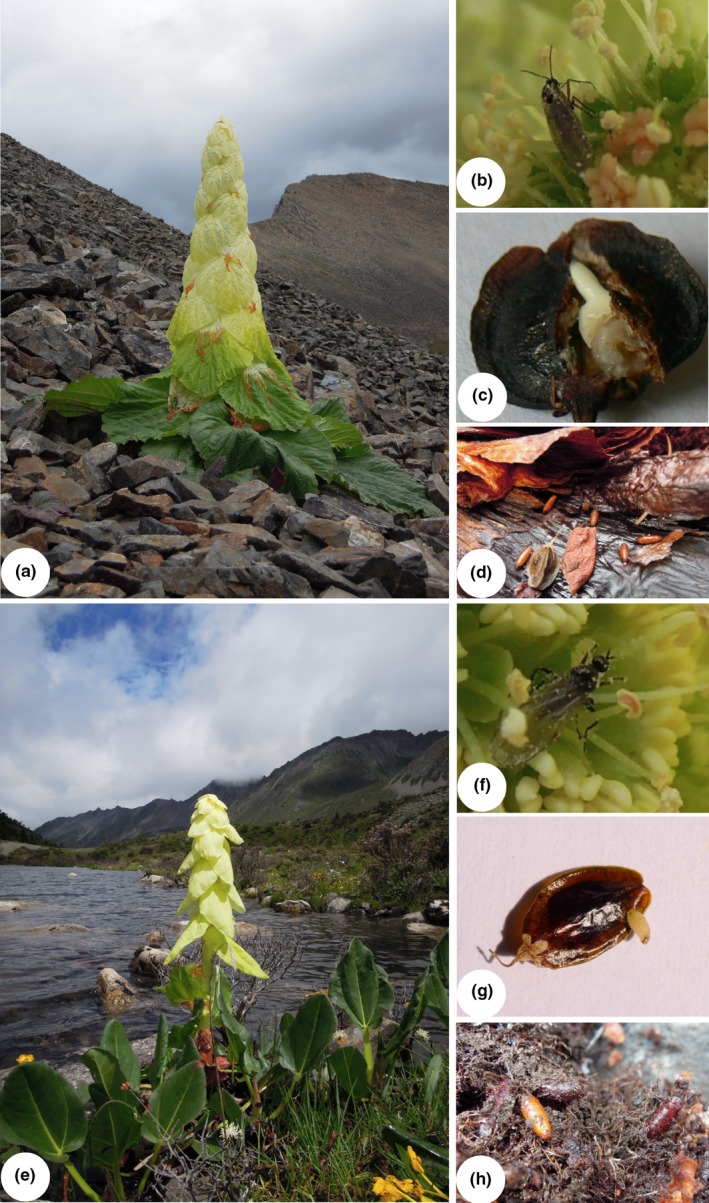
*Rheum nobile* and its pollinator, *Bradysia* sp1. (a–d); *R. alexandrae* and its pollinator, *Bradysia* sp2. (e–h). Flowering plants (a, e), female flies visiting flowers (b, f), fruits infested by fly larvae (c, g), Fly pupae in soil (d, h) (modified from Song et al., 2014, 2015)

Both *Rheum nobile* and *R. alexandrae* are perennial herbs endemic to the high eastern Himalayas, with large translucent cream‐colored bracts covering the entire inflorescence (Figure 1a,e). The two plant species have a clearly distinct distribution associated with difference in soil moisture. *R. nobile* is mainly found on alpine scree, that is, in well‐drained habitats, while *R. alexandrae* usually occurs in alpine wetlands, including marsh, swampy meadows, and lake shores (Song, Stöcklin, et al., 2013). Although the two species are closely related and sympatric throughout part of their distribution range (Song, Stöcklin, et al., 2013; Sun, Wang, Wan, Wang, & Liu, 2012), previous studies confirmed that they are pollinated exclusively by their obligate seed‐consuming fly species, *Bradysia* sp1. and *Bradysia* sp2. (Diptera: Sciaridae), respectively (Figure 1b,f; Song et al., 2014; Song, Stöcklin, Peng, Gao, & Sun, 2015). After feeding on developing seeds in parasite fruits (Figure 1c,g), fly larvae exited from the fruits and burrowed into the soil around the plants (Figure 1d,h), where they overwinter as pupae (Song et al., 2014, 2015). Thus, pupation and adult emergence are completed in the soil. In this study, we investigate whether soil moisture plays a decisive role in driving host specificity of the two fly species. As soil moisture has been identified as a major mortality factor of the soil‐dwelling life stage for most flies (Hulthen & Clarke, 2006; Jackson, Long, & Klungness, 1998; Shililu et al., 2004), we hypothesized that the two fly species are habitat specialists that confine *Bradysia* sp1. to *R. nobile* and *Bradysia* sp2. to *R. alexandrae*. To test this hypothesis, we determined the adult emergence in native and non‐native habitats by conducting a reciprocal transfer experiment in the field. In addition, in the laboratory, we determined (1) the effect of different soil moisture on larval and pupal survival in the two fly species and (2) larval and pupal survival of the two fly species after short‐term submergence in water.

## 2 Materials and Methods

### 2.1 Species description and study sites

*Bradysia* sp1. and *Bradysia* sp2. are the obligate pollinators of the two plant species, *Rheum nobile* and *R. alexandrae*, respectively (Song et al., 2014, 2015). Because of the complex taxonomy of the genus *Bradysia*, the two fly fungus gnats associated with *R. nobile* and *R. alexandrae* are not yet described taxonomically; however, their species difference has been confirmed (YP Wang, personal communication). Both fly species are small insects ranging in body length from 3.5 to 4.3 mm. The two plant species are giant perennial herbs that are endemic to alpine zones (3,000–6,000 m a.s.l.) of the eastern Himalayas, where *R. nobile* is mainly found above 4,000 m a.s.l. and *R. alexandrae* is mainly found above 3,000 m a.s.l. (Li & Gao, 1998). Female flies lay eggs in flowers of their respective host plants and hatched larvae feed on developing seeds. After completing larval growth, fly larvae exit from the fruits and enter the soil to pupate for overwintering.

Larvae of both *Bradysia* species used for experiments were acquired from infested fruits that were collected from at least 25 individuals of each *R. nobile* and *R. alexandrae* in the field and placed on a wire screen. For the field experiments, the wire screens were put in a yard near the study site, while the wire screens were put in the greenhouse for the laboratory experiments. Exiting larvae were collected and were distributed randomly to the different treatments. Laboratory experiments were conducted in a greenhouse at the Yunnan Forestry Technological College (25°05′N–102°46′E, 1,963 m a.s.l.) in Kunming City, Yunnan Province, SW China, with the temperature being kept at 25 ± 2°C in the day and 15 ± 2°C at night. Field experiments were conducted in Huluhai Lake (28°31′N–99°57′E, 4,450 m a.s.l.) in Shangri‐la County, Yunnan Province, SW China, where the species are naturally distributed (see Table 1 for the monthly rainfall recorded between 2012 and 2015 at the nearest meteorological station; 28°23′N, 99°01′E, 4,290 m a.s.l.). For a full description of the study sites, see Song, Zhang, et al. (2013).

**Table 1:** Monthly rainfall (mm) recorded between 2012 and 2015 at a weather station (28°23′N, 99°01′E, 4290 m a.s.l.) nearby the field experimental site

Year	Month											
	1	2	3	4	5	6	7	8	9	10	11	12
2012	19	16	8	22	16	88	133	159	77	32	0	0
2013	1	7	1	38	42	66	137	175	105	35	0	1
2014	5.9	7	5	9.6	7.9	84	184	92.5	36.7	0.8	0	0.6
2015	1.2	1.9	5.9	8.9	13.3	17	84.3	160.9	46.6	41.6	0.1	6.4

### 2.2 Field experiments

#### 2.2.1 Larval dispersal and pupation depth

To determine the dispersal distances by larvae, 80 larvae from each of the two fly species that just exited from their host fruits in the natural population were monitored until they entered the soil and the dispersal distance from their host plant was measured. In addition, in order to determine the soil depth selected by the larvae for pupation, two open ends of iron bottles (15 cm in height and 10 cm in diameter) were carefully inserted into the soil near the plants, without disturbing the soil structure. Ten larvae of each fly species that just exited from their host fruits were released at the surface of each bottle so that they might burrow and then covered with nylon bag. Twenty days later, the soils of each bottle were removed carefully to find the pupae for measuring the depth of pupation in relation to soil surface. Eight replicates of pupation depth were performed for each species.

#### 2.2.2 Reciprocal transfer experiment

To investigate the adult emergence in native and non‐native habitats, a reciprocal transfer experiment was conducted in September, 2014. Twenty open‐bottom pots (15 cm in height and 15 cm in diameter) filled with natural soils were buried at a depth of *c*. 18 cm in each of the habitats of *R. nobile* and *R. alexandrae,* with the soil being naturally compacted under the environmental conditions of the sites. Ten days later, twenty *Bradysia* sp1. larvae that just exited from their host fruits were put on a pot in their native habitat, that is, inhabited by *R. nobile*, and non‐native habitat, that is, inhabited by *R. alexandrae*, respectively. Similarly, twenty *Bradysia* sp2. larvae that just exited from their host fruits were put on a pot in their native habitat and non‐native habitat, respectively, as described above. A 1.8‐cm layer of soil was put on the larvae (corresponding to the optimal pupation depth, see “3”) and then covered with a nylon bag. Each treatment contained 10 pots for each species. In early June 2015, adult emergence was observed by counting and recording the number of adults in the nylon bag every day as the first adult had emerged.

### 2.3 Laboratory experiments

#### 2.3.1 Effect of soil moisture on pupation

In order to test the effect of soil moisture on pupation, 20 larvae of each fly species that just exited from their host fruits were put on the soil surface of pots (15 cm in height and 15 cm in diameter) filled with natural soils and then covered with nylon bag; there were 30 pots with 600 larvae for each species in total. The 30 pots of each species were randomly divided into six groups, and each group of both species was placed into a large plastic container (100 cm in length, 80 cm in width, and 50 cm in height), with each container containing ten pots (five per species). Six soil moisture treatments were established: water level at 5, 0, −5, −10 cm relative to the soil surface in the pots, and dry “a” and dry “b.” For the former four soil moisture treatments, we added water in the containers to reach different water levels relative to the soil surface in the pots. Tap water was supplied daily to maintain the water level. For the two dry treatments, no water was added in the container and pots were watered every eighth day (dry “a”) or fourth day (dry “b”). The six large plastic containers were put closely together under controlled conditions in the greenhouse and could be considered to have same environmental conditions with the exception of water level. Twenty days later, the number of larvae in each pot that had pupated was counted carefully.

In order to determine the effect of soil moisture on depth of pupation, ten larvae of each species were put on the soil surface of a different set of 25 pots and were allowed to burrow and pupate. Experimental conditions were the same as above, but only five soil moisture treatments were performed: water level at 0, −5, −10 cm relative to the soil surface in the pots, dry “a” and dry “b” because no larvae could survive in the 5‐cm treatment (see “3”). Prior to adult emergence, the soils of each pot were removed carefully to search the pupae for measuring the depth of pupation relative to the soil surface. Five replications were performed for each soil moisture treatment.

In order to test the survival of larvae after different time of complete submergence in water, 15 larvae of each fly species that just exited from their host fruits were placed in each of 25 plastic vials (2 cm in diameter and 8 cm in height) open at both ends but covered with nylon mesh to prevent escapes, and the vials were then submerged in water. Subsequently, five vials of each species were taken out every 6 hours and placed into pots provided with the optimal soil water condition (−10 cm; see “3”). Twenty days later, the number of pupae for each treatment was counted carefully.

#### 2.3.2 Effect of soil moisture on emergence

Two thousand larvae of each fly species were placed into a plastic container (100 cm in length, 80 cm in width, and 50 cm in height) containing natural soils and allowed to burrow and pupate. After 20 days, these pupae were taken out and used for the experiments described below. To determine the effect of soil moisture on adult emergence, the six soil moisture treatments described above were set up again in similar pots as used before. Twenty pupae of each species were put on the soil surface of each pot containing natural soils and covered with a layer of soil with different thickness corresponding to the optimal pupation depth at different moisture treatments (see “3”). Each treatment for each species included five replications. Emerged adults were counted every day until there was no increase in adult number for 30 consecutive days.

In order to test the survival of pupa after different time of complete submergence in water, experimental treatments described above were set up again. Fifteen pupae of each fly species were placed in each of 25 plastic vials and submerged in water. Subsequently, five vials of each species were taken out every 6 hours and the pupae were placed into pots provided with the optimal soil water condition (−10 cm) and covered with a layer of soil (*c*. 1.5 cm; see “3”). Emerged adults were counted every day until there was no increase in adult number for 30 consecutive days.

### 2.4 Data analysis

For comparing differences in percentage pupation, percentage emergence and pupation depth of flies among treatments, two‐way ANOVA was used with species and treatment as fixed factors. When a significant interaction between species and treatment was detected, one‐way ANOVA followed by Tukey test (*p *<* *.05) was used in each species in order to distinguish treatment effects. The *Bradysia* sp1. and *Bradysia* sp2. larvae burrowing depth and dispersal distance were compared using independent samples *t*‐tests. All analyses were performed in SPSS 18.0. Measurements are reported as means ± 1 SE.

## 3 Results

### 3.1 Larval dispersal and pupation depth

**Figure 2:**
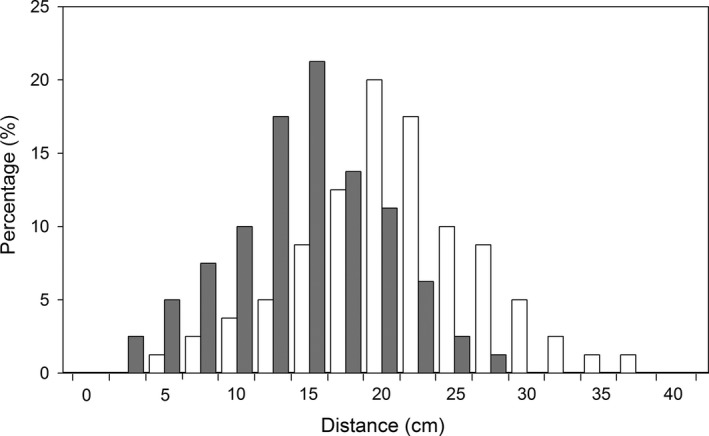
Frequency distribution of dispersal distance of larvae from *Bradysia* sp1. (*dark gray bars*) and *Bradysia* sp2. (*white bars*) after larvae exited from their host fruits in natural field sites

**Figure 3:**
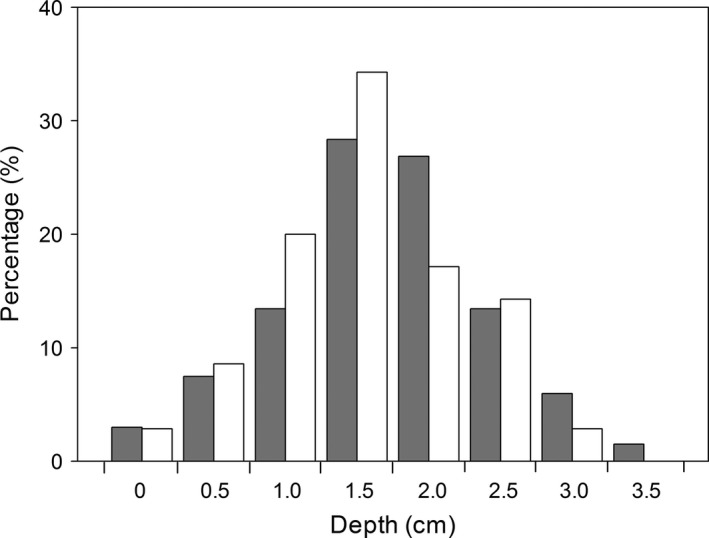
Frequency distribution of pupation depth of *Bradysia* sp1. (*dark gray bars*) and *Bradysia* sp2. (*white bars*) in natural field sites

In the natural population, larvae of both fly species undergo a dispersal episode after exiting from their host fruits. The dispersal distances away from their host plant of most larvae were less than 25 cm for both fly species (Figure 2). The average dispersal distance was larger (19.6 ± 0.7 cm) for larvae of *Bradysia* sp2. than for larvae of *Bradysia* sp1. (13.3 ± 0.6 cm) (*t *=* *6.8, *df *= 158, *p *<* *.001). Most larvae burrowed into natural soil to a depth of 1 to 2.5 cm for pupation for both fly species (Figure 3). There was no difference in the pupation depth between the two species (*t *=* *1.56, *df *= 135, *p *=* *.12), with 1.9 ± 0.09 cm depth for *Bradysia* sp1., and 1.7 ± 0.08 cm depth for *Bradysia* sp2.

### 3.2 Reciprocal transfer experiment

**Figure 4:**
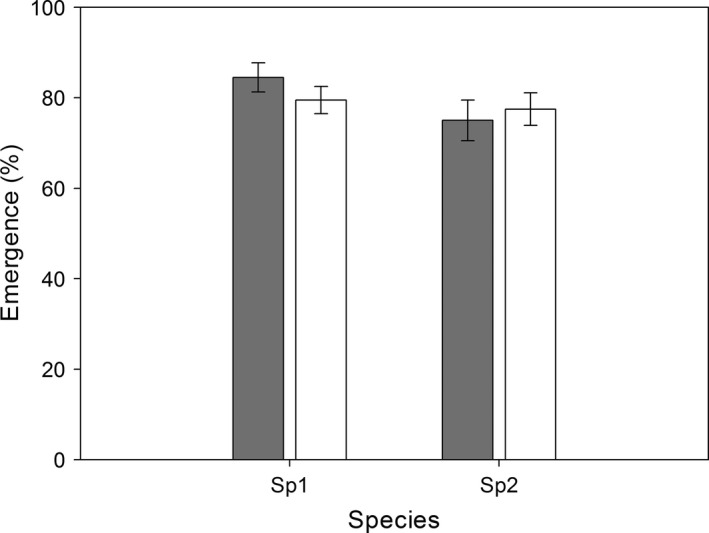
Percentage emergence (Mean ± SE,* n *=* *10) of *Bradysia* sp1. and *Bradysia* sp2. after larvae were placed in their native habitats (*dark gray bars*) and non‐native habitats (*white bars*)

In natural field sites, no difference in the percentage of adult emergence was found between the two species (*F*_1, 36_ **= 2.50, *p *=* *.12; Figure 4). When larvae of *Bradysia* sp1. and *Bradysia* sp2. were separately placed in their native and non‐native habitats, percentage adult emergence was no significantly different for both species (*F*_1, 36_ **= 0.10, *p *=* *.73; Figure 4). Similarly, there was no significant interaction between species and treatment (*F*_1, 36_ **= 1.08, *p *=* *.31, two‐way ANOVA; Figure 4).

### 3.3 Effect of soil moisture on pupation

**Figure 5:**
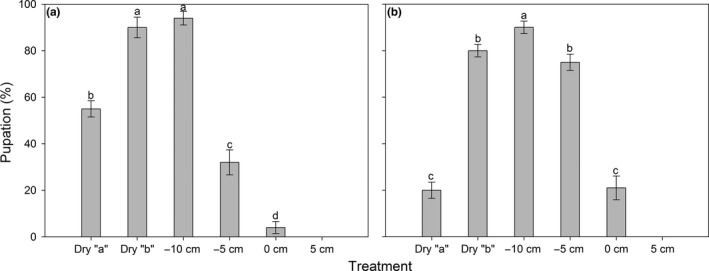
Percentage pupation (Mean ± SE,* n *=* *5) of *Bradysia* sp1. (a) and *Bradysia* sp2. (b) after larvae were placed in different soil moisture treatments. Dry “a” and Dry “b”: watered every eighth day and fourth day, respectively; −10, −5, 0, and 5 cm: water level relative to the soil surface. Different letters indicate significant differences between treatments at *p *<* *.05

No pupae or adults of either species were found in the 5‐cm soil moisture treatment at the end of the experiment, so this treatment was excluded from subsequent analyses. Pupation was significantly affected by the soil moisture treatment (*F*_4, 40_ **= 157.34, *p *<* *.001), and a significant species × treatment interaction was detected (*F*_4, 40_ **= 30.24, *p *<* *.001, two‐way ANOVA), indicating that the effect of treatment on pupation was species‐dependent. The one‐way ANOVA conducted in each species revealed significant effect of treatment on pupation for both fly species (*F*_4, 20_ **= 96.25, *p *<* *.001 and *F*_4, 20_ **= 90.47, *p *<* *.001 for *Bradysia* sp1. and *Bradysia* sp2., respectively). For both species, the maximum percentage of pupation occurred when water level was −10 cm below the soil surface, and percentage of pupation declined with an increase or a reduction in soil water level (Figure 5). However, larvae of *Bradysia* sp1. were clearly more tolerant to drought than *Bradysia* sp2. larvae: In the dry “a” treatment, 55% of larvae for *Bradysia* sp1. pupated, while only 20% of larvae pupated for *Bradysia* sp2. In the water‐logging condition, the opposite result was found: In the 0‐cm treatment, 21% of larvae for *Bradysia* sp2. pupated, while only 4% of larvae pupated for *Bradysia* sp1.

Pupation depth was significantly affected by soil moisture treatment (*F*_4, 40_ **= 758.89, *p *<* *.001), and this effect was similar between the two species (*F*_4, 40_ **= 1.55, *p *=* *.21 for the interaction of species × treatment, two‐way ANOVA). For both species, pupation depth was maximal when soil water level was −10 cm below soil surface, and pupation depth decreased with an increase or a reduction in soil moisture level. In particularly, when water level was at the soil surface (0 cm treatment), this resulted in shallowest pupation depth, with all larvae pupating on the soil surface (Table 2).

**Table 2:** Pupation depth (Mean ± SE, *n *=* *5) of *Bradysia* sp1. and *Bradysia* sp2. after larvae were placed in soil with different moisture level. Dry “a” and Dry “b”: soil watered every eighth day and fourth day, respectively; −10, −5, and 0 cm: water level relative to the soil surface

Treatment	Depth (cm)	
	*Bradysia* sp1.	*Bradysia* sp2.
Dry “a”	0.51 ± 0.03 a	0.49 ± 0.03 a
Dry “b”	1.06 ± 0.04 b	1.13 ± 0.04 b
−10 cm	1.67 ± 0.06 c	1.62 ± 0.04 c
−5 cm	0.84 ± 0.02 d	0.91 ± 0.03 d
0 cm	0 ± 0 e	0 ± 0 e

*Different letters* indicate significant differences between treatments within a species at *p *<* *.05.

**Figure 6:**
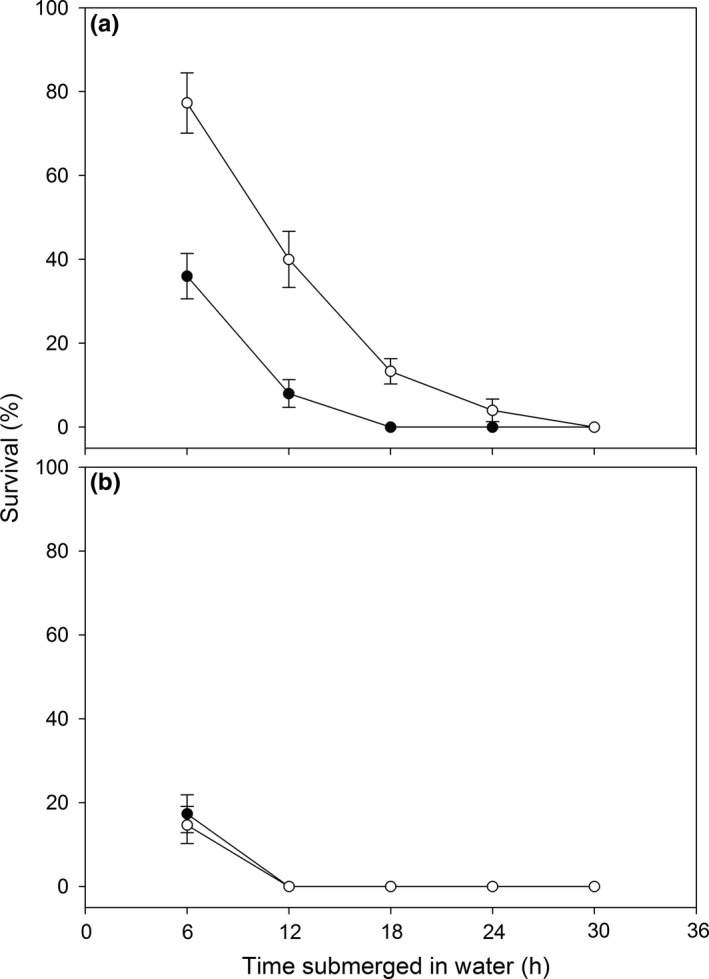
Percentage survival of larvae (a) and pupae (b) of *Bradysia* sp1. (*filled circle*) and *Bradysia* sp2. (*open circle*) after submerged in water for different numbers of hours

The ability of *Bradysia* sp2. larvae to tolerate complete submergence in water was greater than that of *Bradysia* sp1. larvae (Figure 6): with six hours submerged in water, 77% of larvae of *Bradysia* sp2. survived, whereas the equivalent rate was 36% for *Bradysia* sp1. Furthermore, all larvae of *Bradysia* sp2. died after 30 hours submergence in water, while for *Bradysia* sp1., all larvae had died already after 18 hours submergence in water.

### 3.4 Effect of soil moisture on emergence

**Figure 7:**
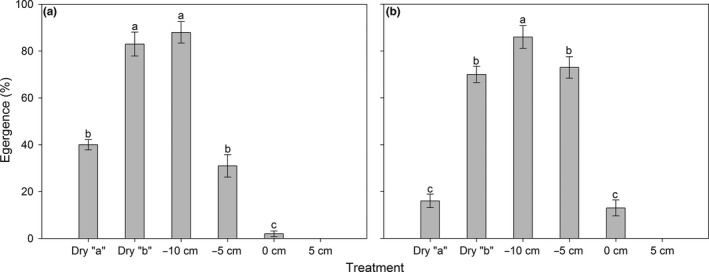
Percentage emergence (Mean ± SE,* n *=* *5) of *Bradysia* sp1. (a) and *Bradysia* sp2. (b) after pupae were placed in different soil moisture treatments. Dry “a” and Dry “b”: watered every eighth day and fourth day, respectively; −10, −5, 0, and 5 cm: water level relative to the soil surface. Different letters indicate significant differences between treatments at *p *<* *.05

Adult emergence was significantly affected by the soil moisture treatment (*F*_4, 40_ **= 140.36, *p *<* *.001), and a significant species × treatment interaction was detected (*F*_4, 40_ **= 20.86, *p *<* *.001, two‐way ANOVA), indicating that the effect of treatment on adult emergence was species‐dependent. The one‐way ANOVA conducted in each species revealed significant effect of treatment on emergence for both fly species (*F*_4, 20_ **= 84.78, *p *<* *.001 and *F*_4, 20_ **= 76.41, *p *<* *.001 for *Bradysia* sp1. and *Bradysia* sp2., respectively). Similar to pupation, the −10‐cm soil water treatment was the most suitable condition for adult emergence in both species, and percentage emergence declined with increasing or decreasing soil moisture level (Figure 7). Pupae of *Bradysia* sp1. was more tolerant to drought than those of *Bradysia* sp2.: In the dry “a” treatment, 40% of pupae for *Bradysia* sp1. emerged, while only 16% of pupae emerged for *Bradysia* sp2. In contrast, in the water‐logging condition (0 cm), 13% of pupae for *Bradysia* sp2. emerged, while only 2% of pupae emerged for *Bradysia* sp1. (Figure 7).

In both species, pupae could tolerate shorter complete submergence in water than larvae (Figure 6). Six hours after being submerged in water, only 17% and 14% of pupae for *Bradysia* sp1. and *Bradysia* sp2., respectively, had survived. Furthermore, pupae of both species submerged in water did not survive beyond 12 hours.

## 4 Discussion

According to the preference/performance hypothesis (Thompson, 1988), if the environmental conditions of oviposition sites affect offspring performance, the difference in environmental conditions may play an important role in choice of host plants by adult insects (König et al., 2016). As a result, there should be a good correspondence between oviposition preference and offspring performance. Similar to previous reports on the effects of soil moisture on performance of larva–pupal stage in some insects (Hou et al., 2006; Hulthen & Clarke, 2006; Jackson et al., 1998; Vargas, Chang, Komura, & Kawamoto, 1987), our laboratory experiments showed that soil moisture did have significant effects on larval and pupal performances in both species and extremely wet or dry soils significantly decreased their survival. However, the two fly species had similar optimal soil moisture requirements for both pupation and adult emergence. Moreover, our field reciprocal transfer experiment also revealed that adult emergence was not affected by the transfer to the habitat of another species, indicating that offspring perform equally well in their native and non‐native habitats and female oviposition preference was not correlated with larval–pupal performance in terms of soil moisture for both fly species. This means that soil moisture is unlikely to be the primary reason for host specificity of the two *Bradysia* species. Recently developed feeding niche constraints hypothesis may help to explain such lack of positive correlation between oviposition preference and larval–pupal performance (Craig & Itami, 2008; Gripenberg et al., 2010; Soto, Goenaga, Hurtado, & Hasson, 2012). It has been argued that habitat complexity may weaken the preference–performance relationship, as the change in feeding niche between oviposition and completion of immature development may limit the female's ability to predict the quality of resources for larval and pupal development (Craig & Itami, 2008; Gripenberg et al., 2010). For example, if a larva must move to complete development, the cues available at the oviposition site may have very little predictive value about the quality of microhabitat for pupation and adult emergence (Craig & Itami, 2008). This might be the case of the two *Bradysia* species as their eggs are laid in flowers whereas pupation and adult eclosion take place in soils around the host plants (Song et al., 2014, 2015). In addition, season variation in rainfall because of prevailing monsoon climate in alpine zone of the eastern Himalayas may aggravate the habitat complexity in terms of soil moisture. Thus, ovipositing females seem to be unlikely to predict future soil moisture conditions that will be experienced by their larvae and pupae.

Despite the fact that its host, *R. alexandrae*, mainly occurs in wetlands, pupation and emergence of *Bradysia* sp2. were significantly inhibited by extremely wet soils under controlled conditions. The pupation and emergence characteristics of *Bradysia* sp2. may be an adaptation to the soil moisture conditions during the period in the year when pupation and emergence are occurring. Pupation of *Bradysia* sp2. occurs in late September and early October and adult emergence commences in early June, when the rainy season has ended or has not yet started, respectively. So, the natural water level at the time of the year when pupae and emergence are occurring is very low, especially in seasonal wetlands (Song, Stöcklin, et al., 2013; Wang, Li, Wang, & Chen, 2007). For example, the water level of Huluhai Lake in the dry season is almost 40 cm lower than in the rainy season.

Soil moistures were found to influence pupation depth of both fly species under controlled conditions: deepest pupation occurred at intermediate water levels and depth decreased with a reduction or an increase in soil water level, which is in agreement with many other studies (Dimou, Koutsikopoulos, Economopoulos, & Lykakis, 2003; Jackson et al., 1998; Tsitsipis & Papanicolaou, 1979). A plausible explanation is that low soil moistures negatively affect the vitality and activity of the larvae. For example, due to water evaporation, soils with low moisture content caused a hard surface and *Bractrocera* larvae could only burrow to a relatively shallow depth (Dimou et al., 2003), while a high soil moisture content can aggravate oxygen deficit in deep soil layers and induce shallow or surface pupation (Dimou et al., 2003; Norsworthy & Oliveira, 2006). It is worth noting that shallow pupation due to moisture extremes might leave larvae and pupation more vulnerable to predation and less protected from desiccation and freezing, which may further increase the mortality of larvae and pupation (Renkema, Cutler, Lynch, MacKenzie, & Walde, 2011).

Interestingly, even though larval survival of both species was inhibited by high soil moisture levels, their larvae could survive temporarily flooding. Furthermore, larvae of *Bradysia* sp2. were more tolerant to flooding than *Bradysia* sp1., which is of advantage in their nature habitat and could be an adaptation to these conditions. Plants of *R. alexandrae*, host of *Bradysia* sp2., are sometimes surrounded by water; in this case, after exiting from the fruits, larvae necessarily fall into the water. Thus, the ability of *Bradysia* sp2. larvae to survive short‐term submergence in water, in combination with their higher dispersal ability, may allow them to move to suitable pupation sites. Similar to a previous study on another fly species, pupae responded differently to the short‐term submergence in water than larvae, experiencing higher mortality rates than larvae (Eskafi & Fernandez, 1990). In addition, although like in *Bradysia* sp2., pupation and emergence were inhibited by drought stress, *Bradysia* sp1. was more tolerant to drought than *Bradysia* sp2., probably because the habitats inhabited by *Bradysia* sp1. are more vulnerable to drought events. These results indicate that in the two fly species, pupation and adult emergence in response to extremely wet or dry soils are habitat‐specific. Using pupation and adult emergence as proxies for larval and pupal survivals could be questioned because larvae of some fly species could regulate development through dormancy, even, constructing spherical cocoons to avoid unfavorable soil moisture conditions (Readshaw, 1968). However, in our study, all unpupated larvae and unemerged pupae of both species had decayed, or exhibited evidence of mortality (e.g., waterlogging and fungal growth). Thus, the drastical reduction in pupation and adult emergence under extremely dry or wet soil moistures should be due to larval or pupal death, not of dormancy.

Evidence from our experiments suggests that soil moisture condition is unlikely to play a determining role in regulating host specificity of the two fly species. Nevertheless, the different tolerances of *Bradysia* sp1. and *Bradysia* sp2. to flooding or drought stress were habitat‐specific: The species occurring in well‐drained habitats is more tolerant to drought stress than the species occurring in wetlands, while the species occurring in wetlands is more tolerant to flooding stress than the species occurring in well‐drained habitats. It appears likely that the two fly species are confined to their respective host plants for other reasons first, and then have adapted to the specific habitat conditions associated with soil moisture level. Further studies testing the probable role of host plant nutritional acceptability, secondary plant defenses and morphological constraints in host specificity of the two *Bradysia* species should be conducted to understand the evolution of the obligate pollination mutualism in the two *Rheum* species (Bernays & Graham, 1988).

## Conflict of Interest

None declared.

## References

[ece32644-bib-0001] Arvanitis, L. , Wiklund, C. , & Ehrlén, J. (2007). Butterfly seed predation: effects of landscape characteristics, plant ploidy level and population structure. Oecologia, 152, 275–285. 17479297 10.1007/s00442-007-0659-5

[ece32644-bib-0002] Arvanitis, L. , Wiklund, C. , & Ehrlén, J. (2008). Plant ploidy level influences selection by butterfly seed predators. Oikos, 117, 1020–1025.

[ece32644-bib-0003] Bernays, E. , & Graham, M. (1988). On the evolution of host specificity in phytophagous arthropods. Ecology, 69, 886–892.

[ece32644-bib-0004] Bonebrake, T. C. , Boggs, C. L. , McNally, J. M. , Ranganathan, J. , & Ehrlich, P. R. (2010). Oviposition behavior and offspring performance in herbivorous insects: Consequences of climatic and habitat heterogeneity. Oikos, 119, 927–934.

[ece32644-bib-0005] Bressan‐Nascimento, S. (2001). Emergence and pupal mortality factors of *Anastrepha oblique* (Macq.)(Diptera:Tephritidae) along the fruiting season of the host *Spondias dulcis* L. Neotropical Entomology, 30, 207–215.

[ece32644-bib-0006] Chen, M. , & Shelton, A. M. (2007). Impact of soil type, moisture, and depth on Swede midge (Diptera: Cecidomyiidae) pupation and emergence. Environmental Entomology, 36, 1349–1355. 18284762 10.1603/0046-225x(2007)36[1349:iostma]2.0.co;2

[ece32644-bib-0007] Chew, F. S. , & Robbins, R. K. (1984). Egg‐laying in butterflies. Symposium of Royal Entomological Society of London, 11, 65–79.

[ece32644-bib-0008] Courtney, S. P. (1986). The ecology of pierid butterflies: Dynamics and interactions. Advances in Ecological Research, 15, 51–131.

[ece32644-bib-0009] Craig, T. P. , & Itami, J. K. (2008). Evolution of preference and performance relationship In Tilmon K. J. (Ed.), Specialization, speciation, and radiation. The evolutionary biology of herbivorous insects (pp. 20–28). Berkeley, CA: University of California Press.

[ece32644-bib-0010] Dempster, J. P. (1983). The natural control of populations of butterflies and moths. Biological Reviews, 58, 461–481.

[ece32644-bib-0011] Dimou, I. , Koutsikopoulos, C. , Economopoulos, A. P. , & Lykakis, J. (2003). Depth of pupation of the wild olive fruit fly, *Bactrocera* (Dacus) *oleae* (Gmel.) (Dipt., Tephritidae), as affected by soil abiotic factors. Journal of Applied Entomology, 127, 12–17.

[ece32644-bib-0012] Ehrlich, P. R. , & Raven, P. H. (1964). Butterflies and plants: A study in coevolution. Evolution, 18, 586–608.

[ece32644-bib-0013] Eskafi, F. M. , & Fernandez, A. (1990). Larval‐pupal mortality of Mediterranean fruit fly (Diptera: Tephritidae) from interaction of soil, moisture, and temperature. Environmental Entomology, 19, 1666–1670.

[ece32644-bib-0014] Fry, J. D. (1996). The evolution of host specialization: Are trade‐offs overrated? American Naturalist, 148, 84–107.

[ece32644-bib-0015] Gripenberg, S. , Mayhew, P. J. , Parnell, M. , & Roslin, T. (2010). A meta‐analysis of preference‐performance relationships in phytophagous insects. Ecology Letters, 13, 383–393. 20100245 10.1111/j.1461-0248.2009.01433.x

[ece32644-bib-0016] Hou, B. , Xie, Q. , & Zhang, R. (2006). Depth pupation and survival of the Oriental fruit fly, *Bactrocera dorsalis* (Diptera: Tephritidae) pupae at selected soil moistures. Applied Entomology and Zoology, 41, 515–520.

[ece32644-bib-0017] Hulthen, A. D. , & Clarke, A. R. (2006). The influence of soil type and moisture on pupal survival of *Bactrocera tryoni* (Froggatt) (Diptera: Tephritidae). Australian Journal of Entomology, 45, 16–19.

[ece32644-bib-0018] Jackson, C. G. , Long, J. P. , & Klungness, L. M. (1998). Depth of pupation in four species of fruit flies (Diptera: Tephritidae) in sand with and without moisture. Journal of Economic Entomology, 91, 138–142.

[ece32644-bib-0019] Jaenike, J. (1990). Host specialization in Phytophagous insects. Annual Review of Ecology and Systematics, 21, 243–273.

[ece32644-bib-0020] Janzen, D. H. (1979). How to be a fig. Annual Review of Ecology and Systematics, 10, 13–51.

[ece32644-bib-0021] Johnson, S. N. , Zhang, X. X. , Crawford, J. W. , Gregory, P. J. , & Young, I. M. (2007). Egg hatching and survival time of soil‐dwelling insect larvae: A partial differential equation model and experimental validation. Ecological Modelling, 202, 493–502.

[ece32644-bib-0022] Joshi, A. , & Thompson, J. N. (1995). Trade‐offs and the evolution of host specialization. Evolutionary Ecology, 9, 82–92.

[ece32644-bib-0023] Kato, M. , Takimura, A. , & Kawakita, A. (2003). An obligate pollination mutualism and reciprocal diversification in the tree genus *Glochidion* (Euphorbiaceae). Proceedings of the National Academy of Sciences, 100, 5264–5267. 10.1073/pnas.0837153100 PMC154333 12695568

[ece32644-bib-0024] König, M. A. E. , Wiklung, C. , & Ehrlén, J. (2016). Butterfly oviposition preference is not related to larvae performance on a polyploidy herb. Ecology and Evolution, 6, 2781–2789. 27217940 10.1002/ece3.2067 PMC4863005

[ece32644-bib-0025] Li, A. R. , & Gao, Z. J. (1998). Flora of China. Beijing: Science Press.

[ece32644-bib-0026] Martin, L. A. , & Pullin, A. S. (2004). Host‐specialisation and habitat restriction in an endangered insect, *Lycaena dispar bataus* (Lepidoptera: Lycaenidae) II. Larvae survival on alternative host plant plants in the field. European Journal of Entomology, 101, 57–62.

[ece32644-bib-0027] Montoya, P. , Flores, S. , & Toledo, J. (2008). Effect of rainfall and soil moisture on survival of adults and immature stages of *Anastrepha ludens* and *A. oblique* (Diptera: Tephritidae) under semi‐field conditions. Florida Entomologist, 91, 643–650.

[ece32644-bib-0028] Norsworthy, J. K. , & Oliveira, M. J. (2006). Sicklepod (*Senna obtusifolia*) germination and emergence as affected by environmental factors and seedling depth. Weed Science, 54, 903–909.

[ece32644-bib-0029] Pellmyr, O. , Thompson, J. N. , Brown, J. M. , & Harrison, R. G. (1996). Evolution of pollination and mutualism in the yucca moth lineage. American Naturalist, 148, 827–847.

[ece32644-bib-0030] Rausher, M. D. (1984). Tradeoffs in performance on different hosts: Evidence from within‐ and between‐site variation in the beetle *Deloyala guttata* . Evolution, 38, 582–595. 10.1111/j.1558-5646.1984.tb00324.x 28555973

[ece32644-bib-0031] Readshaw, J. L. (1968). Damage to Swedes by the swede midge, *Contarinia nasturtii* (Kieff.), and a possible method of cultural control. Bulletin of Entomological Research, 58, 25–29.

[ece32644-bib-0032] Renkema, J. M. , Cutler, G. C. , Lynch, D. H. , MacKenzie, K. , & Walde, S. J. (2011). Mulch type and moisture level affect pupation depth of *Rhagoletis mendax* Curran (Diptera: Tephritidae) in the laboratory. Journal of Pest Science, 84, 281–287.

[ece32644-bib-0033] Shililu, J. I. , Grueber, W. B. , Mbogo, C. M. , Githure, J. I. , Riddiford, L. M. , & Beier, J. C. (2004). Development and survival of Anopheles gambiae eggs in drying soil: Influence of the rate of drying, egg age, and soil type. Journal of the American Mosquito Control Association, 20, 243–247. 15532921

[ece32644-bib-0034] Singer, M. C. (2000). Reducing ambiguity in describing plant‐insect interactions: “performance”, “acceptability” and “electivity”. Ecology Letters, 3, 159–162.

[ece32644-bib-0035] Slansky, F. J. (1993). Nutritional ecology: The fundamental quest for nutrients In Stamp N. E., & Casey T. E. (Eds.), Ecological and evolutionary constraints on foraging (pp. 29–91). New York, NY: Chapman & Hall.

[ece32644-bib-0036] Song, B. , Chen, G. , Stöcklin, J. , Peng, D. L. , Niu, Y. , Li, Z. M. , & Sun, H. (2014). A new pollinating seed‐consuming mutualism between *Rheum nobile* and a fly fungus gnat, *Bradysia* sp., involving pollinator attraction by a specific floral compound. New Phytologist, 203, 1109–1118. 24861151 10.1111/nph.12856

[ece32644-bib-0037] Song, B. , Stöcklin, J. , Gao, Y. Q. , Zhang, Z. Q. , Yang, Y. , Li, Z. M. , & Sun, H. (2013). Habitat‐specific responses of seed germination and seedling establishment to soil water condition in two Rheum species in the high Sino‐Himalayas. Ecological Research, 28, 643–651.

[ece32644-bib-0038] Song, B. , Stöcklin, J. , Peng, D. L. , Gao, Y. Q. , & Sun, H. (2015). The bracts of the alpine ‘glasshouse’ plant *Rheum alexandrae* (Polygonaceae) enhance reproductive fitness of its pollinating seed‐consuming mutualist. Botanical Journal of the Linnean Society, 179, 349–359.

[ece32644-bib-0039] Song, B. , Zhang, Z. Q. , Stöcklin, J. , Yang, Y. , Niu, Y. , Chen, J. G. , & Sun, H. (2013). Multifunctional bracts enhance plant fitness during flowering and seed development in *Rheum nobile* (Polygonaceae), a giant herb endemic to the high Himalayas. Oecologia, 172, 359–370. 23124332 10.1007/s00442-012-2518-2

[ece32644-bib-0040] Soto, E. M. , Goenaga, J. , Hurtado, J. P. , & Hasson, E. (2012). Oviposition and performance in natural hosts in cactophilic Drosophila. Evolutionary Ecology, 26, 975–990.

[ece32644-bib-0041] Sun, Y. S. , Wang, A. L. , Wan, D. S. , Wang, Q. , & Liu, J. Q. (2012). Rapid radiation of *Rheum* (Polygonaceae) and parallel evolution of morphological traits. Molecular Phylogenetics and Evolution, 63, 150–158. 22266181 10.1016/j.ympev.2012.01.002

[ece32644-bib-0042] Thompson, J. N. (1988). Evolutionary ecology of the relationship between oviposition preference and performance of offspring in phytophagous insects. Entomologia Experimentalis et Applicata, 47, 3–14.

[ece32644-bib-0043] Tsitsipis, J. A. , & Papanicolaou, E. P. (1979). Pupation depth in artificially reared olive fruit‐flies *Dacus oleae* (Diptera: Tephritidae), as affected by several physical characteristics of the substrates. Annales de zoologie, ecologie animale, 11, 31–40.

[ece32644-bib-0044] Vargas, R. I. , Chang, H. B. , Komura, M. , & Kawamoto, D. (1987). Mortality, stadial duration and weight loss in three species of mass‐reared fruit fly pupae (Diptera: Tephritidae) held with and without vermiculite at selected relative humidities. Journal of Economic Entomology, 80, 972–974.

[ece32644-bib-0045] Wang, G. X. , Li, Y. S. , Wang, Y. B. , & Chen, L. (2007). Typical alpine wetland system changes on the Qinghai‐Tibet Plateau in recent 40 years. Acta Geographica Sinica, 62, 481–491.

[ece32644-bib-0046] Whittaker, R. H. , & Feeny PP (1971). Allelochemicals: Chemical interactions between species. Science, 171, 757–770. 5541160 10.1126/science.171.3973.757

[ece32644-bib-0047] Yang, P. , Li, Z. B. , Peng, Y. Q. , & Yang, D. R. (2012). Exchange of hosts: Can agaonid fig wasps reproduce successfully in the figs of non‐host *Ficus*? Naturwissenschaften, 99, 199–205. 22271213 10.1007/s00114-012-0885-5

